# Nitrous oxide use and psychiatric disorders: a retrospective clinical cohort study on prevalence and patterns

**DOI:** 10.3389/fpsyt.2025.1670500

**Published:** 2025-10-01

**Authors:** Rebecca Paetow, Maike Franziska Dohrn, Michelle Finner-Prével, Leona Boesehans, Dariush Henning, Marcus Rust, Thomas Frodl

**Affiliations:** ^1^ Department of Psychiatry, Psychotherapy and Psychosomatics, RWTH Aachen University, Aachen, Germany; ^2^ Department of Neurology, RWTH Aachen University, Aachen, Germany; ^3^ Deutsches Zentrum Psychische Gesundheit, Center for Intervention and Research on adaptive and maladaptive brain Circuits underlying mental health (CIRC) Jena, Magdeburg, Germany

**Keywords:** nitrous oxide, psychiatric disorders, substance use, lifetime prevalence, ADHD, depression, cannabis-related disorders, novel psychoactive substance

## Abstract

**Introduction:**

Nitrous oxide (N_2_O) is used for anesthetic purposes but has gained popularity as a recreational substance. Despite its potentially severe adverse effects, knowledge about N_2_O use within psychiatric populations is limited. This study aimed to evaluate the life-time prevalence and patterns of N_2_O consumption among patients with psychiatric disorders.

**Methods:**

A retrospective observational cohort study was conducted at the Department of Psychiatry, Psychotherapy and Psychosomatics of the Rheinisch-Westfälische Technische Hochschule (RWTH) Aachen University Hospital, involving assessments of N_2_O use lifetime prevalence among patients in various psychiatric settings over a six-month period in 2024. Further data on demographic characteristics and psychiatric diagnoses were collected from electronic patient records.

**Results:**

Out of 287 screened records, 22 patients (7.67%) reported a N_2_O use history, with a positive statistical relationship between younger age and positive lifetime prevalence (mean age: 28.14 ± 7.29 years, range 19–48 years, 6/22 female). Most users acquired N_2_O through low-threshold means such as friends or social events. The predominant psychiatric diagnoses among users included major depressive disorder, cannabis-related disorder and attention deficit and hyperactivity disorder.

**Discussion:**

This study highlights the concerning life-time prevalence of N_2_O use in a clinical psychiatric sample, emphasizing the need for increased awareness and education regarding its potential risks and side effects. Given the vulnerability of this population to substance-related issues, routine assessment for N_2_O use should be integrated into standard psychiatric evaluations.

## 1 Introduction

Nitrous oxide (N_2_O) is a color-less gas that is commonly used for anesthetic procedures in the fields of dental and emergency medicine (1, 2). This substance is an NMDA receptor antagonist comparable to ketamine (3). Its first use as an anesthetic agent was reported in 1884 by the American dentist Horace Wells (4). Nearly a century earlier in 1772, N_2_O was first synthesized and described by Joseph Priestley. In the late 1790s, Humphry Davy reported its psychotropic properties (5). By the early 1800s, this effect led to the public use of N_2_O for entertainment purposes (see (2) for a historical overview). Today, N_2_O is used in the automotive industry for performance vehicles and in the culinary industry in whipped cream dispensers, also called whippets, besides its use in the medical field for short-term anesthesia (6).

**Figure 1:**
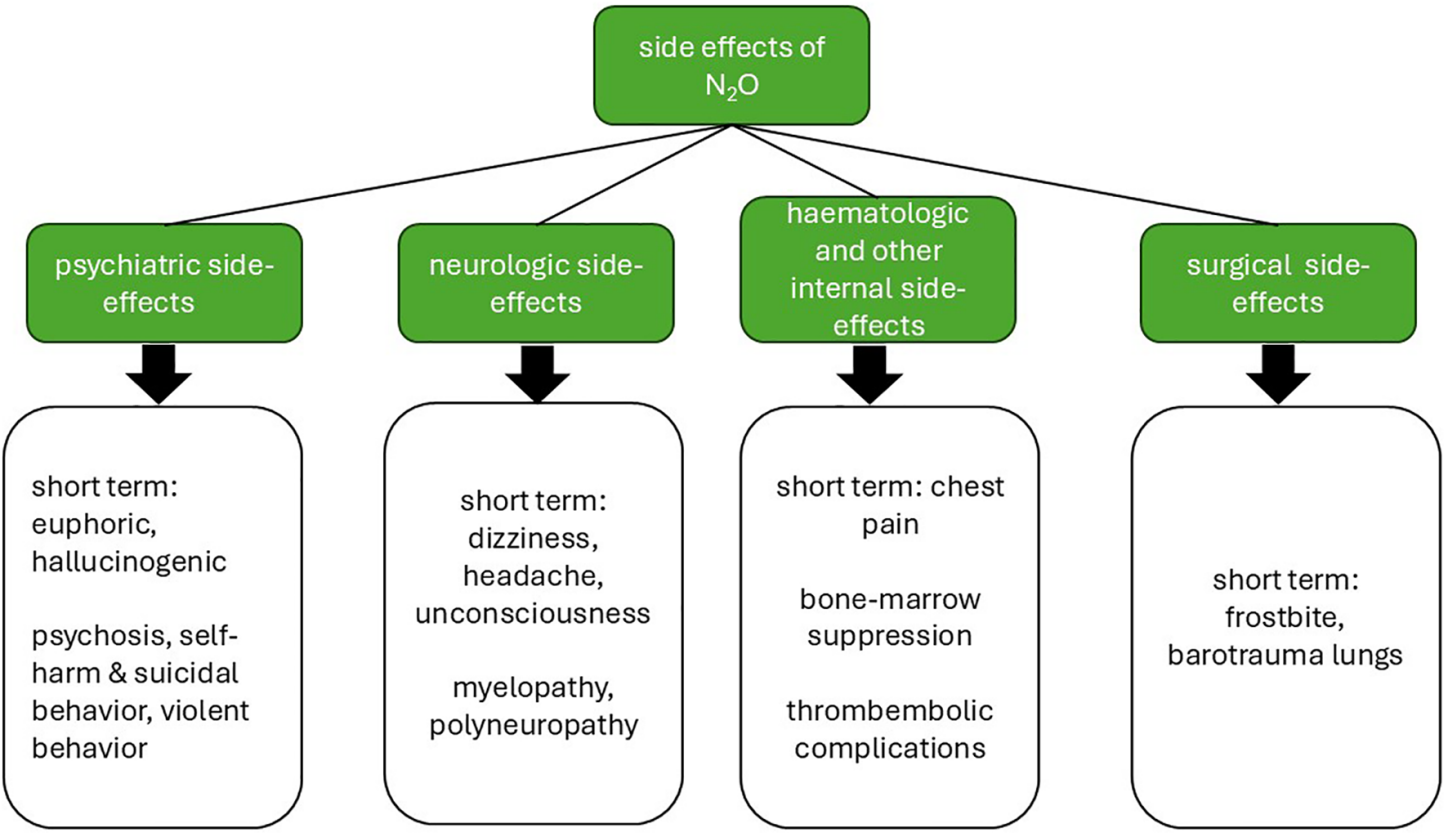
Overview of side-effects of N_2_O use by medical fields. Illustration created by the authors with Microsoft PowerPoint.

N_2_O is easily accessible as a so-called Novel Psychoactive Substance (NPS) (7). A NPS is a substance that produces psychoactive effects similar to controlled drugs but is legally available, non-detectable in routine drug screenings and therefore allegedly considered safe by the general public (8). The widespread availability of NPS, for example in online markets or public sales, makes them attractive for recreational use, especially among young people. This phenomenon has been described especially for the use of N_2_O. For Europe in July 2014, the European Court of Justice ruled that products without an apparent therapeutic effect cannot be classified as a medical product leading to a low threshold of selling N_2_O to the public (9). The Global Drug Survey of 2014 described a concerning rise in the recreational use of N_2_O in the time span of 12 months (6). N_2_O is often supplied either from small cartridges (see above “whippets”) or large tanks. The gas is then transferred to balloons and subsequently inhaled (10). Short-term effects cover auditory and visual hallucinations and a euphoric high lasting for up to two minutes (11, 12). Besides this intended effect, adverse side-effects on different organ systems have been reported for N_2_O -consumption (see **Figure 1**). Acute side-effects comprise dizziness, headache, unconsciousness or chest pain (13). Inhaling N_2_O under high pressure can also cause barotrauma of the lungs and emphysema (14). Skin contact with N_2_O tanks while filling a ballon can lead to frostbite (15). N_2_O consumption can also cause psychosis, self-harming and suicidal or violent behavior (11, 16). The most important adverse side-effect complex of N_2_O is caused by a disturbance of cobalamin metabolism inactivating vitamin B12. However, toxicity of N_2_O may not be solely linked to inactivating vitamin B12 as some patients display normal vitamin B12 levels (17). This can lead to severe neurological and hematopoietic side-effects like polyneuropathy, or even myelopathy, thromboembolic complications and bone-marrow suppression (18–23). In most scientific reports, severe side-effects have been linked to chronic and high dose consumption of N_2_O (6, 20). Supplementation of vitamin B12 combined with discontinuation of N_2_O consumption can potentially reverse toxic side-effects (24).

Patterns of consumption can be broadly categorized into heavy and non-heavy use. In context of a Dutch cohort study, heavy use is defined by the number of balloons (≥ 50 balloons) or the weight (≥ 400 g) or the volume (≥ 200 L) consumed per session (9). Chronic and heavy N_2_O consumption has been linked to severe adverse effects, and some authors debate whether it fulfills the diagnostic criteria for substance dependency (13).

N_2_O has a short half-life of approximately 5 minutes (25) and does not appear to act on dopaminergic pathways like classic addictive substances (26, 27). Case reports show that some individuals engage in repetitive and long-term use (28–30), suggesting a potential for dependency. Given that patients with psychiatric diagnoses constitute a particularly vulnerable group, evaluating N_2_O use within this population is crucial. Previous research – as described above - has primarily investigated N_2_O use in the general population, case reports, clinical management of adverse effects, its potential for addiction and biochemical mechanisms. A large-scale national survey study in England demonstrated a significant association between substance use and higher rates of psychological morbidity (31). Therefore, further investigation into the specific pattern of N_2_O use in psychiatric samples is needed. This retrospective study addresses this gap by presenting one of the first analyses of lifetime prevalence of N_2_O use in a representative psychiatric sample.

## 2 Methods

### 2.1 Setting and study design

The Department of Psychiatry, Psychotherapy and Psychosomatics at Rheinisch-Westfälische Technische Hochschule Aachen (RWTH) University Hospital uses a default assessment for first patient contact. This assessment includes standardized questions for past substance use. After in-house training, the assessment was expanded to include past contact with N_2_O at the day-hospital, outpatient clinic and two inpatient wards. The day-hospital specializes in patients with mood disorders, psychotic disorders, obsessive-compulsive disorder, anxiety disorders and posttraumatic stress disorder. The outpatient clinic focuses on hyperactivity deficit and attention disorders (ADHD), autism, mood disorders, dementia, sleep disorders and further differential diagnostics. The first in-patient ward specializes in treating substance use disorders (SUD, “SUD-ward”), the second in treating psychotic disorders (“psychosis-ward”). Both wards also provide crisis interventions in an open setting, for example for suicidal crises independent of an underlying psychiatric disorder. All patients were at least 18 years old due to regulation of admission.

For this retrospective observational cohort study, patient charts were evaluated over a six-month period in 2024 (January to June in the polyclinic and May to October in day-hospital and inpatient wards). This study was approved by the local ethics committee (internal reference numbers EK 24-459, CTC-A-Number 24-523) including a waiver for the requirement for signed consent due to the retrospective nature of the study and use of anonymized data for analyses.

### 2.2 Assessment of lifetime prevalence of N_2_O use

In context of local and national news coverage, an internal clinical training session was held on interviewing patients about N_2_O use. The two questions “Have you ever been exposed to N_2_O, also called laughing gas, before?” and “Have you ever used N_2_O outside of a clinical context?” were asked and then followed by a short and open-ended interview in case of affirmation of the second question. The aim was to gain a general overview of the life-time prevalence of consumption. These semi-structured interviews were conducted by the authors RP, MFD, MFP, LB, DH and MR. All patients were asked during admission or shortly after admission to each respective department. Each interview was accompanied by an explanation of risks, side-effects and pathomechanisms of N_2_O. Previously, the N_2_O survey was not part of the routine drug screening carried out by the clinic. In Germany, screening for N_2_O is currently not part of the standard routine in emergency departments or in neurological and psychiatric clinics. Increasing numbers of cases with neurological complications have been reported in major cities in Germany (32, 33). As a result, clinical awareness is rising and N_2_O is assessed more frequently when suspicious clinical presentations occur.

### 2.3 Variables and outcomes

The primary outcome was the life-time prevalence for N_2_O use outside of a clinical context in the cohort. The secondary outcomes were group differences between patients with or without past consumption for age and gender plus distribution of International Statistical Classification of Diseases and Related Health Problems, 10th Revision (ICD-10, 34), diagnoses and further reported characteristics of N_2_O consumption and psychiatric history in the group with past consumption.

### 2.4 Data sources and analysis

The following data from electronic patient records were anonymized and transferred to a database in Microsoft Excel: age, gender (male, female, non-binary), mode of presentation (inpatient, outpatient and day-hospital), past life-time N_2_O use outside of medical context (yes, including further anamnestic context if given, or no), psychiatric diagnoses according to ICD-10 and further descriptive information about referral and prior psychiatric history. Patients usually received more than one diagnosis. Statistical analysis was conducted with Microsoft Excel (35) and R (36).

Age was reported with mean, standard deviation and range. A Chi-square-test (statistical significance set at *p <.05*) was implemented for the relationship between N_2_O use and gender and a Welch t-test (statistical significance set at *p <.05*) on age in relation to N_2_O use. For stratification of age by gender and by mode of presentation, additional Welch t-tests were performed.

## 3 Results

### 3.1 Participants

287 records were screened in total (93 outpatient, 115 inpatient and 79 day-hospital) after exclusion of 16 records due to missing assessment.

### 3.2 Descriptive data

Mean age of the outpatient cohort was 34.89 ± 11.99 years (range 19–77 years), 43.65 ± 14.35 years (range 18–85 years) for the inpatient cohort and 35.41 ± 14.59 years (range 19–76 years) for the day-hospital cohort. Stratification by gender revealed no significant age differences within the three modes of presentation. For further demographic data, please refer to **Table 1**. A detailed breakdown of age by mode of presentation and gender is provided in **Supplementary Table S3**.

**Table 1:** Demographic data by mode of presentation for age, gender and past N_2_O consumption.

Characteristic	Out-patient (n = 93)	In-patient (n = 115)	Day-hospital (n = 79)
Age (mean ± SD)	34.89 ± 11.99	43.65 ± 14.35	35.41 ± 14.59
Male	43	72	41
Female	50	43	37
Non-binary	0	0	1
Past N_2_O consumption	10	6	6

Columns are divided by admission type, with the number of patients indicated (n = …).

### 3.3 Primary and secondary outcomes

The prevalence of past life-time N_2_O use was 7.67% (n = 22), including 10 outpatients, 6 inpatients (n = 3 for the SUD-ward and the psychosis-ward, respectively), and 6 day-hospital patients. There was no significant gender difference in consumption, *χ²* (2) = 3.27, *p* = .195. It should be noted that all female patients with past life-time N_2_O use (n = 6) were out-patients. Patients with past nitrous oxide consumption were significantly younger than those without, *t* (36.49) = -6.31, *p* <.001 (28.14 ± 7.29 years versus 39.41 ± 14.38 years). When t-tests were performed separately for female/male gender, the age difference persisted (female: *t* (7.15) = 3.42, *p* = .01 (28.00 ± 6.54 years versus 38.00 ± 13.19 years); male *t* (30.67) = 5.40, *p* <.001 (28.19 ± 7.75 years versus 40.76 ± 15.29 years).

Single-time use was reported by 10 patients and two patients reported discontinuation of consumption due to unpleasant side-effects. Most patients reported a low-threshold acquisition (n = 8) and consumption at parties or other social events (n = 9). Usage years ago was reported by 9 patients. Please refer to **Table 2** for an overview of anamnestic aspects of prior N_2_O consumption. Only one patient (see patient number 10 in **Supplementary Table S1**) could be considered fulfilling diagnostic criteria for a period of constant use of N_2_O with repeated regular use. Detailed information for all patients with positive history for past N_2_O consumption can be seen in the **Supplementary Tables S1** and **S2**.

**Table 2:** Retrospective categorization of anamnestic aspects of prior nitrous oxide consumption; multiple responses (n > 1) per survey possible.

Description	N times reported
Single use	10
Repeated use	5
Low-threshold acquisition (supermarket, internet, whipped cream charger)	8
At parties or at other social events	9
Discontinued use due to unpleasant side-effects	2

Most patients with prior consumptions were referred for diagnostics (n = 12) or therapeutic stabilization (n = 7). More than 50% (n = 12) of patients with prior consumption reported distressing psychiatric symptoms since their youth or adolescence. All patients (n = 22) with prior consumption had at least one previous contact with a psychiatric institution. Most of them were referred by an outpatient psychiatrist (n = 11) or general practitioner (n = 5). (Detailed information for all patients with positive history for past N_2_O consumption can be seen in the **Supplementary Table S2**).

**Figure 2:**
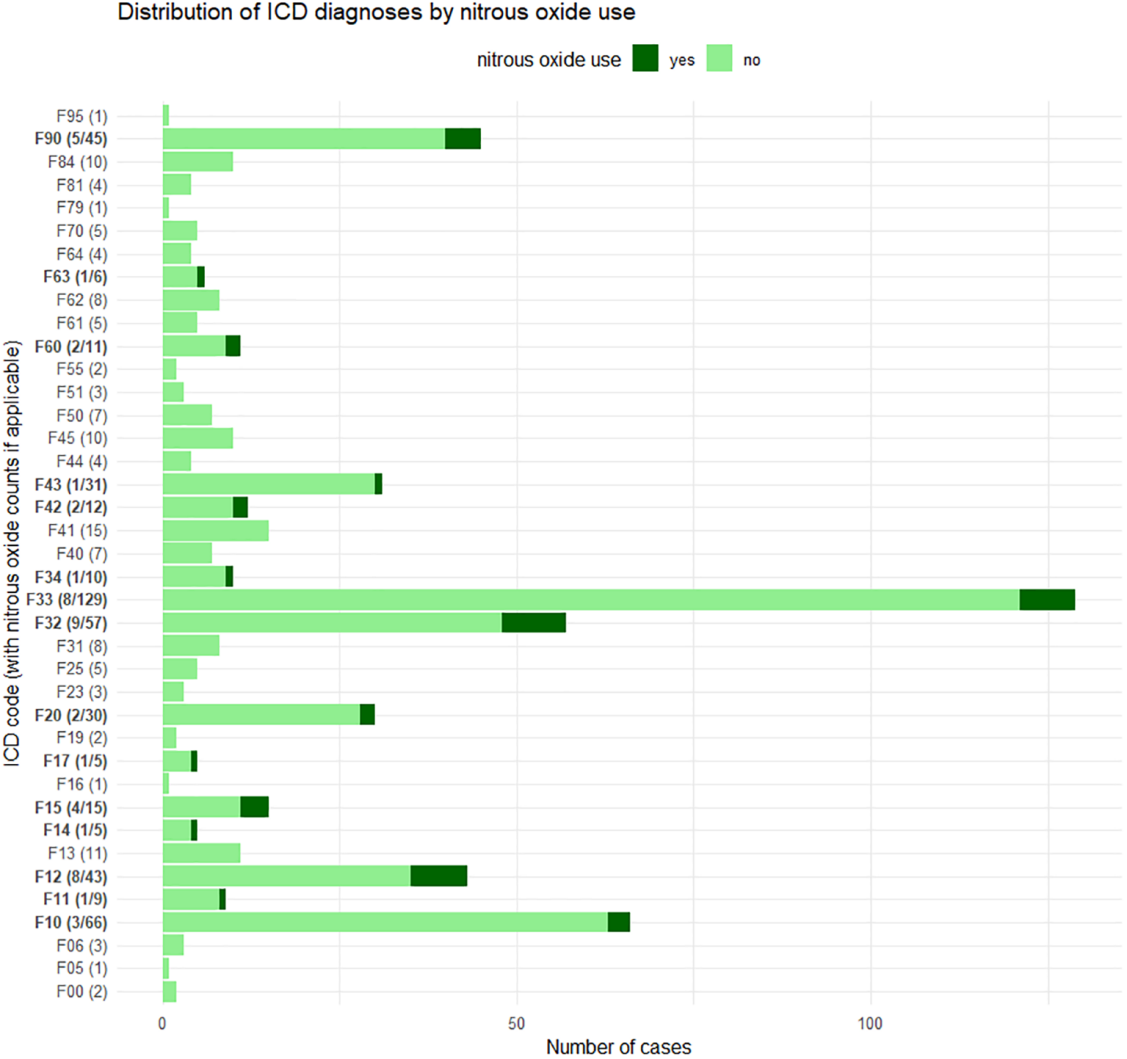
Diagnoses (F-diagnoses according to ICD-10) with past nitrous oxide use and corresponding proportion of all patients surveyed. Multiple diagnoses per survey are possible. Bars are labeled with *yes/total* only if nitrous oxide use occurred in that diagnosis category. Diagnoses included: F00 dementia in Alzheimer’s disease, F05 delirium not induced by alcohol and other psychoactive substances, F06 other mental disorders due to brain damage and dysfunction and to physical disease, F10 alcohol use disorder, F11 opioid use disorder, F12 cannabis use disorder, F13 sedative or hypnotic use disorder, F14 cocaine use disorder, F15 stimulant use disorder, F16 hallucinogen use disorder, F17 tobacco use disorder, F19 polysubstance use disorder, F20 schizophrenia, F23 acute transient psychotic disorders, F25 schizoaffective disorder, F31 bipolar affective disorder, F32 depressive episode, F33 recurrent depressive disorder, F34 persistent mood [affective] disorders, F40 phobic disorders, F41 other anxiety disorders, F42 obsessive–compulsive disorder, F43 stress-related and adjustment disorders, F44 dissociative [conversion] disorders, F45 somatoform disorders, F50 eating disorders, F51 nonorganic sleep disorders, F55 harmful use of non-dependence-producing substances, F60 specific personality disorders, F61 mixed and other personality disorders, F62 enduring personality changes, F63 habit and impulse disorders including media addiction, F64 gender identity disorders, F70 mild intellectual disability, F79 unspecified mental disability, F81 scholastic developmental disorders, F84 pervasive developmental disorders, F90 hyperkinetic disorders, and F95 tic disorders.

Among patients with a positive history of past N_2_O consumption, the most frequent diagnoses in absolute numbers were depression (n = 8 with recurrent depression; n = 9 with a depressive episode), cannabis use disorder (n = 8), and hyperactivity and attention disorder (n = 5). When considering relative frequencies within each diagnostic group of all patients surveyed, the highest proportions of past N_2_O consumption were found among patients diagnosed with tobacco use disorder (1/5), cocaine use disorder (1/5), and other stimulant use disorders (4/15). There were no patients with diagnosed polyvalent substance abuse and reports of past N_2_O consumption. In general, there were no patients diagnosed with F18 (volatile solvent use disorder). An overview of ICD-10 psychiatric diagnoses, including absolute and relative frequencies, can be seen in **Figure 2**.

After final chart review in February 2025, there were 23 records where the diagnostic procedures were still ongoing (18 records with pending diagnostics for hyperactivity and attention disorder, 3 records with pending diagnostics for autism spectrum and 2 records with pending diagnostics for trauma). For patients with positive lifetime prevalence, there were no pending diagnostics.

## 4 Discussion

### 4.1 Summary of main results

This study showed that a relevant portion of patients (7.67%) of a psychiatric service had at least once consumed N_2_O in their past life-time. Patients with positive life-time prevalence of N_2_O substance use were significantly younger with a mean age of 28.14 years. Most of the patients reported a use in a social context like parties or festivals and easy accessibility of N_2_O receiving it via friends or acquaintances. Moreover, most patients reported single use. For psychiatric diagnosis, there was an overrepresentation of depression (recurrent or first episode), mental or behavioral disorders due to the use of cannabinoids and ADHD.

### 4.2 Age-related patterns

In previous studies, usage of N_2_O has often been reported with an association to adolescent and younger age. A narrative review from 2022 (37) with data from Europe, the USA, Australia and Asia reported a relevant and partially rising trend of N_2_O use in young people. For example, in the Netherlands there was an increase from 20.8% to 25.2% in the age-group of 20 to 24 years in the use prevalence between 2016 and 2018. A representative German survey among adolescent students (15 to 18 years) reported a rising trend in substance history of N_2_O until 2017 and a falling, but still relevant trend afterwards with 9% in 2019 (38). This emphasis on younger individuals might be caused by N_2_O being a relatively novel substance. Information on its use is widely shared through the internet and social media making it more accessible to younger audiences (39, 40). Despite this emphasis on younger individuals, the prevalence of N_2_O use in adults and most of all older cohorts in Western and Asian countries remains unclear. Currently, cross-sectional epidemiological data on N_2_O use across all age groups is insufficiently recorded. A systematic review from 2022 on recreational N_2_O use including 34 studies (original research, literature reviews and case reports) reported over half of all study participants under age 30 and only two large surveys with participants up to age 60 (41). This gap underlines the relevance of the findings of the present study in an adult population, considering the psychiatric vulnerability and broad age range represented. Further studies and surveys are needed to better characterize the age distribution of N_2_O use efficiently.

### 4.3 Global perspective on N_2_O availability

A low-threshold accessibility, as reported by most patients in our sample, aligns with previous findings. From a recent historical perspective, N_2_O entered the European scene as a NPS before expanding to broader social settings. For example, a British review from 2016 (2) described N_2_O as a common recreational drug at festivals and university parties in the United Kingdom before it was there defined as an illegal drug in 2023 (42). A European report further describes use in open public places, at home, pre-clubbing parties and at so called-car parties (usage with friends in a parking lot) (43). Due to a rapid rise in recreational use and public health implications, several European countries have implemented restrictions. For example, in the United Kingdom and the Netherlands laughing gas is considered as an illegal drug (42, 44). In Germany, the 128^th^ German Medical Assembly 2024 called for a ban on sales to minors and strict regulation on general public sales (45). France, Ireland and Denmark have prohibited public sales to people below the age of 18. However, this can be probably circumvented by online access (46). In the United States, N_2_O use has likewise emerged as a relevant medical concern (47). Reported use increases by 58% between 2023 and 2024. The Sale of small canisters (e.g. “Galaxy Gas”) remains largely unregulated and the expansion of recreational use is further facilitated by loopholes in current American legislation.

### 4.4 N_2_O, psychiatry comorbidity and problematic substance use

Individuals with psychiatric disorders represent a vulnerable group for problematic substance use. A well-documented relationship exists between problematic substance use and other psychiatric conditions, particularly mood disorders such as depression and bipolar disorder (48). In a review from 2022 about comorbidity between mood and substance-related disorders including 120 studies, there was a fivefold risk between depression and cannabis dependence and a sixfold elevated risk between broadly defined mood-disorders and substance-related disorders in general (49). Hypotheses explaining these associations include the self-medication theory (50) and overlapping neurobiological pathways, especially in the mediating role of the nucleus accumbens, an important brain region for behavioral regulation (51). In a large, representative Australian survey from 2017 (n = 36,309), cannabis use disorder was strongly associated with the use of illicit drugs, prescription medications, and stimulant-based substances, including club drugs. The data were derived from the National Epidemiologic Survey on Alcohol and Related Conditions (52). This review discussed a multi-structured system of personal and socio-economic factors as a possible underlying cause (52).

ADHD is an established risk factor for substance use disorders and its development in adulthood (53). Children and adolescents with ADHD are more likely to use alcohol, tobacco, and illicit substances than their peers without ADHD (54). The underlying mechanisms remain unclear, though theories suggest that executive dysfunction and impulsiveness contribute to increased substance use vulnerability (55). Increased impulsivity combined with heightened sensation seeking in ADHD could contribute to misuse of N_2_O as a fast-acting psychoactive substance. Another explanation might be self-medication in ADHD. According to a 2025 study (56), individuals with ADHD are more likely to use NPS for self-medication. This has been linked to difficulties in accessing adequate treatment and diminished confidence in healthcare services.

In line with these broader findings, the present cohort revealed an overrepresentation of individuals with depression, mental and behavioral disorders due to substance use and ADHD. In absolute terms, depression was the most frequent diagnosis among individuals with past N_2_O use, followed by cannabis use disorder and ADHD. In terms of relative prevalence within diagnostic categories, the highest proportions of past N_2_O use were observed among patients with tobacco use disorder, cocaine use disorder and other stimulant use disorders. This is consistent with the increased risk for problematic substance use as described above. Notably, only life-time prevalence data on N_2_O use was collected. Nevertheless, given the well-established susceptibility of individuals with psychiatric disorders to substance-related problems, emerging substances like N_2_O call for attention and identification of both early indicators for problematic use and dependency.

### 4.5 Association with other substance use disorders

The high prevalence of past N_2_O use among patient with tobacco use disorder, cocaine use disorder and other stimulant use disorders might be explained by a tendency towards polydrug abuse, even though no past N_2_O use was identified among those diagnosed with polyvalent substance abuse. In general, tobacco has been called a so-called gateway drug (57). Particularly adolescents who smoke tobacco are at increased risk of polydrug use (58). Another possible explanation is the overlap of consumption settings, since cocaine and other stimulants are also prevalent in the party scene (59), like N_2_O. In this context, a poison center report from Michigan with 144 cases reported a 30% ratio of polysubstance involvement in N_2_O incidents (60).

### 4.6 Study limitations

To the best of current knowledge, this is one of the first representative studies exploring life-time prevalence of N_2_O use in a large adult psychiatric cohort. Some limitations must be considered. The main limitation of this study is a possible selection bias. As a monocentric and retrospective cohort study, initial presentation of patients from specific units has been assessed for 6 months. Since the chosen units focus on specific disorders, e.g. substance use and psychotic disorders, the percentage of prior N_2_O consumption with these diagnoses appears to be low. It should be noted that diagnostic categories were not represented equally. For instance, diagnoses such as tobacco (F17) and cocaine use disorders (F14) were underrepresented compared to other substance-related or affective disorders. Prevalence with those diagnosed with depression was high among patients with positive history of prior N_2_O consumption. No specific affective disorder ward was included, and the included outpatient clinic does not focus on depression. The high rate of individuals with depression may represent a selection bias: patients with affective disorders might have been more likely to seek professional care, even in institutions not primarily focusing on mood disorders. Additionally, patients with major depressive disorder might have applied N_2_O as self-medication due to its antidepressant effect (3, 61). Another bias might be introduced by assessment strategy of life-time prevalence: A semi-structured short interview was conducted to assess prior N_2_O consumption apart from medical use and any further information provided by the patient was documented. This further information was not structured but explored openly. Open-ended questions, in contrast to standardized interviews, could have introduced inter-interviewer variability and memory bias by patients. The interview did not contain standardized questions for quantity and frequency of consumption. It did not contain a standardized assessment of neurological, psychiatric or other clinical side-effects of N_2_O consumption or clinical management in case of report of adverse effects, either. This limits the assessment of dose-dependent risk of adverse effects, past clinical management and the general temporal relationship between psychiatric diagnosis and N_2_O consumption. A cause-effect relationship or a possible impact of N_2_O consumption on the onset or worsening of psychiatric disorders cannot be estimated by the given study design. Besides, there is a potential underreporting in lifetime-prevalence since patients might not have been truthful about prior N_2_O consumption. There is no drug test for N_2_O outside of controlling for vitamin B12, homocysteine and methylmalonic acid levels in a blood test (11).

### 4.7 Future directions and implications for clinical practice

Future research should explore longitudinal trajectories of N_2_O use and investigate potential risk factors for progression from occasional to problematic use. Long-term observational studies in clinical and population-based settings are needed to identify patterns of N_2_O use escalation and identify individual, social and clinical risk factors. On-site standardized assessment of previous N_2_O consumption will be implemented. A structured assessment of frequency, duration, dosage, side-effects and previous supplementation of vitamin B12 is recommended (for a best practice recommendation please refer to **Supplementary Table S4**). Additionally, neurobiological mechanism underlying susceptibility for using N_2_O frequently should be foremost in further investigations. Therapeutical implications include a heightened sensitivity in psychiatric exploration and a standardized assessment of N_2_O usage, including medical education about side-effects and risks. Given the potential for neurological, internal, surgical, and psychiatric adverse effects, even in the absence of frequent use, early identification in adolescents and young adults is essential. This is particularly relevant for patients with depression, ADHD, or substance use disorders, and should be accompanied by education provided by healthcare professionals.

### 4.8 Conclusion

In summary, a relevant life-time prevalence of prior N_2_O history in a clinical psychiatric sample was observed. Since N_2_O is a novel and commonly-used drug with severe and underestimated side-effects, there is a strong need for a rise in awareness and education for patients and health care providers. Asking for N_2_O life-time, past-year and past-month prevalence and informing about mechanisms and side-effects across all age groups should be part of standard operating procedures for psychiatric assessments since psychiatric patients form a vulnerable pool.

## Data Availability

The datasets presented in this article are not readily available because of confidentiality restrictions. Requests to access the datasets should be directed to the corresponding author.
