# Supplementary material for: Nitrous oxide use and psychiatric disorders: a retrospective clinical cohort study on prevalence and patterns
Source: Front Psychiatry. 2025 Oct 1;16:1670500. doi: 10.3389/fpsyt.2025.1670500 (PMC12522402; doi:10.3389/fpsyt.2025.1670500)
Supplement: Supplementary file 1 [file SupplementaryFile1.docx]

**Supplement S1:** **Detailed information for patients with positive life-time prevalence for N_2_O consumption including admission type, diagnosis, age, gender and free responses on N_2_O use**

| **Number** | **Admission type** | **Diagnoses from chapter V (F) of the ICD-10** | **Age range in years** | **Gender** | **Free responses on N_2_O use** |
| --- | --- | --- | --- | --- | --- |
| 1 | out-patient, ADHD assessment | F32 | 18 - 24 | w | single contact with N_2_O (at a friend´s place, procurement method unclear) |
| 2 | out-patient, ADHD assessment | F90, F33 | 25 – 34 | m | purchased in a supermarket |
| 3 | out-patient, ADHD assessment | F32 | 35 – 44 | m | tried N_2_O at a party in a nearby European country about 9 years ago |
| 4 | out-patient, ADHD assessment | F32 | 18 – 24 | w | single contact with N_2_O, accessed through a group of friends, consumption from ballons; developed headaches during the process and therefore stopped further use; patient was informed about potential risks of N_2_O |
| 5 | out-patient, ADHD assessment | F33, F15, F12, F90 | 35 – 44 | w | experimented with N_2_O (once at a party), cocaine, ecstasy, and ketamine 10-15 years ago |
| 6 | out-patient, ADHD assessment | F32, F90 | 25 – 34 | w | approximately 5 past contacts with N_2_O; each time with friends, who obtained a large canister in a nearby European country; patient was informed about potential risks |
| 7 | out-patient, ADHD assessment | F90, F32 | 25 – 34 | m | Consumed N_2_O several times at festivals and parties; ordered from a widely accessible online retailer; last use about 3 years ago; patient was informed about potential harm |
| 8 | out-patient, ADHD assessment | (Re-testing in clinical neuropsychology was recommended since ADHD was not reliably ruled out) | 25 – 24 | w | single contact with N_2_O at a activity group for adolescents in 2018; consumed from whipped cream chargers |
| 9 | out-patient, ADHD assessment | F32 | 18 – 24 | w | approximately once a year N_2_O use; obtained from friends or online; patient was informed about risks |
| 10 | out-patient, ADHD assessment | F32 | 25 – 34 | m | repeated regular use of N_2_O (every two weeks); ordered online; long break of consumptions during a biographically challenging life phase, single use again the day before initial presentation |
| 11 | in-patient, SUD ward | F11, F12 | 18 – 24 | m | single use approximately 2 years ago |
| 12 | in-patient, SUD ward | F10, F33, F12 | 18 – 24 | m | single N_2_O use in 2023 as a party drug at a party in a proximate metropolitan area; due to poor tolerance, the patient refrained from further use; had previously received N_2_O once as an anesthetic during a dental procedure |
| 13 | in-patient, SUD ward | F33, F12 | 25 – 34 | m | occasional N_2_O use as an intoxicant; also received it as an anesthetic during medical procedures |
| 14 | day-clinic | F33, F60, F43, F10, F15 | 25 – 34 | m | (No further information provided) |
| 15 | day-clinic | F42, F12 | 18 – 24 | m | in the past, he also used N_2_O as an intoxicant but discontinued after learning about side-effects |
| 16 | day-clinic | F12, F17, F14 | 18 – 24 | m | single use of N_2_O from a ballon at a party 5 years ago |
| 17 | day-clinic | F33, F34 | 18 – 24 | m | N_2_O use as an intoxicant approximately 3 to 4 times before the COVID pandemic |
| 18 | day-clinic | F33 | 45 + | m | single use in 2019 at a party during a trip to Southeast Asia |
| 19 | day-clinic | F33, F42, F90 | 18 – 24 | m | single N_2_O use for its effect on the voice, presumably not for intoxicating purposes |
| 20 | in-patient, psychosis ward | F32, F12 | 18 – 24 | m | tried N_2_O at most twice; did not like it |
| 21 | in-patient, psychosis ward | F20, F12, F63, F15, F10 | 35 – 44 | m | tried N_2_O once at a party 3-4 years ago from a whipped cream charger |
| 22 | in-patient, psychosis ward | F20, F15, F50, F32 | 25 – 34 | m | used N_2_O 3 times at a party a few years ago |

**Supplement S2:** **Detailed information for patients with positive life-time prevalence for N_2_O for further information about referral and previous history**

| **Number** | **Self-referral?** | **From whom they where referred?** | **Reason of referral** | **Duration of psychiatric illness** | **Previous contact to other psychiatric service?** |
| --- | --- | --- | --- | --- | --- |
| 1 | no | outpatient psychiatrist | ADHD diagnostics | describes distressing symptoms back to school days | yes (outpatient psychiatrist) |
| 2 | no | general practitioner with psychotherapeutic training | ADHD diagnostics | Exact duration unclear, previous repeated treatments (in-patient, day-hospital),  psychotherapeutic treatment by general practitioner, no specialist psychiatric treatment | yes (inpatient, day clinic, outpatient) |
| 3 | no | outpatient psychiatrist | ADHD diagnostics | describes distressing symptoms back to school days | yes (outpatient) |
| 4 | no | outpatient psychiatrist | ADHD diagnostics | describes distressing symptoms back to school days | yes (outpatient psychotherapy, rehabilitation program) |
| 5 | no | outpatient psychiatrist | ADHD diagnostics | first distressing symptoms and contact with drugs at the age of 13 | yes (outpatient psychotherapy) |
| 6 | no | general practitioner | ADHD diagnostics | describes distressing symptoms back to school days | yes (outpatient psychotherapy) |
| 7 | no | outpatient psychiatrist | ADHD diagnostics | describes distressing symptoms back to school days | yes (outpatient psychotherapy) |
| 8 | no | outpatient psychiatrist | ADHD diagnostics | describes distressing symptoms back to school days | yes (outpatient psychotherapy) |
| 9 | no | outpatient psychiatrist | ADHD diagnostics | describes distressing symptoms back to school days | yes (outpatient psychotherapy) |
| 10 | no | outpatient psychiatrist | ADHD diagnostics | describes distressing symptoms back to school days | yes (outpatient psychotherapy) |
| 11 | no | general practitioner | qualified withdrawal from opioids | use of cannabis and opioids since the age of 19 | no (refugee status) |
| 12 | yes | emergency admission on-site | qualified withdrawal from alcohol | depression for three years, excessive alcohol consumption for 7 months | yes (outpatient psychotherapy) |
| 13 | yes | emergency admission on-site | exacerbation of depression, suicidal thought | depression for seven years | yes (outpatient psychotherapy) |
| 14 | no | psychiatric ward on-site | further affective stabilization | diagnosis of borderline personality disorder in 2019 | yes (outpatient psychotherapy, inpatient) |
| 15 | no | outpatient psychiatrist | treatment of advanced obsessive-compulsive disorder | pathological obsessive-compulsive symptoms since primary school | yes (outpatient psychiatrist and psychotherapy) |
| 16 | no | external outpatient clinic | Stabilization in case of risk of chronification of substance-related addictions and psychosis | symptoms of schizophrenia for one year and pathological drug use since October 2023 | yes (outpatient psychotherapy, outpatient psychiatrist, occupational therapy) |
| 17 | yes | patient request | stabilization in advanced depression | depression for two to three years | yes (outpatient psychotherapy) |
| 18 | no | outpatient psychiatrist | stabilization in advanced depression | depression for four years | yes (inpatient, outpatient psychiatrist, outpatient psychotherapy) |
| 19 | no | outpatient psychiatrist | stabilization in advanced depression and obsessive-compulsive disorder | treatment of depression in a child and youth facility | yes (day clinic, outpatient psychiatrist) |
| 20 | no | general practitioner | diagnosis and stabilization for suspected schizophrenia | symptoms of schizophrenia for one year | yes (inpatient, outpatient psychiatrist) |
| 21 | yes | emergency admission on-site | panic attack due to acute psychosis | first psychotic episode at the age of early adulthood | yes (inpatient, outpatient psychiatrist, long-term addiction treatment) |
| 22 | no | general practitioner | diagnosis and stabilization for suspected schizophrenia | addiction problem since later adolescence | yes (inpatient, outpatient psychiatrist) |

**Supplement S3: Age distribution by presentation mode and gender. No significant age differences were observed between male and female patients across the three modes of presentation*****. One patient from day-hospital identified as non-binary and is not included in the table for reasons of anonymization.**

|  | **Female out-patient (n = 50)** | **Male out-patient (n = 43)** | **Female in-patient (n = 43)** | **Male in-patient (n = 72)** | **Female day-hospital (n = 37)** | **Male day-hospital (n = 41)** |
| --- | --- | --- | --- | --- | --- | --- |
| Age (mean ± SD) | 33.84 ± 10.98 | 36.12 ± 13.08 | 44.65 ± 12.50 | 43.06 ± 15.41 | 34.27 ± 13.39 | 36.68 ± 15.74 |
| Past N_2_O consumption | 6 | 4 | 0 | 6 | 0 | 6 |

* out-patient: *t* (82.40) = - 0.90, *p* = .37, in-patient: *t* (102.73) = 0.61, *p* = .55, day-hospital: *t* (75.75) = - 0.73, *p* = .47

**Supplement S4: Best practice Recommendation for anamnestic N_2_O assessment**

| **History, frequency and duration?** | At what age was N_2_O first used?  How often and at what intervals is N_2_O used per week/ month/ year? |
| --- | --- |
| **Dosage?** | Non-heavy Use   - < 50 balloons or - < 400 g or - < 200 L of N_2_O consumed per session   Heavy Use   - ≥ 50 balloons or - ≥ 400 g or - ≥ 200 L of N_2_O consumed per session |
| **Side-effects?** | Psychiatric   - Short term: Euphoric or hallucinogenic? - Signs of psychosis? - Self-harming or suicidal behavior? - Violent behavior?   Neurologic   - Short term: Dizziness, headache or unconsciousness? - Signs of myelopathy? - Signs of polyneuropathy?   Haematologic/ internal   - Short term: Chest pain? - Signs of bown-marrow suppression? - Thrombembolic complications?   Surgical   - Short term: Frostbite? Signs of Barotrauma? |
| **Supplementation of vitamin B12?** | Was vitamin B12 previously supplemented independently or within a professional medical setting?  How was it administered (orally, intravenously, intramuscularly)?  At what dosage and frequency was supplementation administered? |
